# Pregnant Women Develop a Specific Immunological Long-Lived Memory Against SARS-COV-2

**DOI:** 10.3389/fimmu.2022.827889

**Published:** 2022-02-10

**Authors:** Claudio Fenizia, Irene Cetin, Davide Mileto, Claudia Vanetti, Irma Saulle, Maria Di Giminiani, Marina Saresella, Francesca Parisi, Daria Trabattoni, Mario Clerici, Mara Biasin, Valeria Savasi

**Affiliations:** ^1^ Department of Pathophysiology and Transplantation, University of Milan, Milan, Italy; ^2^ Department of Biomedical and Clinical Sciences, University of Milan, Milan, Italy; ^3^ Department of Woman, Mother and Neonate Buzzi Children’s Hospital, ASST Fatebenefratelli‐Sacco, Milan, Italy; ^4^ Clinical Microbiology, Virology and Bio-emergence Diagnosis, ASST Fatebenefratelli-Sacco, Milan, Italy; ^5^ Unit of Obstetrics and Gynecology, ASST Fatebenefratelli-Sacco, Milan, Italy; ^6^ Fondazione don Carlo Gnocchi, IRCCS, Milan, Italy

**Keywords:** SARS-CoV-2, pregnancy, immunological memory, antibody, long term

## Abstract

It is well established that pregnancy induces deep changes in the immune system. This is part of the physiological adaptation of the female organism to the pregnancy and the immunological tolerance toward the fetus. Indeed, over the three trimesters, the suppressive T regulatory lymphocytes are progressively more represented, while the expression of co-stimulatory molecules decreases overtime. Such adaptations relate to an increased risk of infections and progression to severe disease in pregnant women, potentially resulting in an altered generation of long-lived specific immunological memory of infection contracted during pregnancy. How potent is the immune response against SARS-CoV-2 in infected pregnant women and how long the specific SARS-CoV-2 immunity might last need to be urgently addressed, especially considering the current vaccinal campaign. To address these questions, we analyzed the long-term immunological response upon SARS-CoV-2 infection in pregnant women from delivery to a six-months follow-up. In particular, we investigated the specific antibody production, T cell memory subsets, and inflammation profile. Results show that 80% developed an anti-SARS-CoV-2-specific IgG response, comparable with the general population. While IgG were present only in 50% of the asymptomatic subjects, the antibody production was elicited by infection in all the mild-to-critical patients. The specific T-cell memory subsets rebalanced over-time, and the pro-inflammatory profile triggered by specific SARS-CoV-2 stimulation faded away. These results shed light on SARS-CoV-2-specific immunity in pregnant women; understanding the immunological dynamics of the immune system in response to SARS-CoV-2 is essential for defining proper obstetric management of pregnant women and fine tune gender-specific vaccinal plans.

## Introduction

It is widely recognized that pregnancy prompts deep changes in the immune system (1). This is part of the physiological adaptation of the female organism to the pregnancy and the immunological tolerance toward the fetus (2). However, such adaptation results in a possible increased risk of infections and progression to severe disease in pregnant women (3–7). As pregnancy progresses, hormones concentration varies dramatically, and the immune response undergoes a number of still only partially understood para-physiologic modifications (8–10). Thus, pregnancy is associated with a Th1/Th2 shift (11–13), but also in an altered generation of long-lived specific immunological memory towards infective pathogens contracted during pregnancy. In fact, pregnancy has short- and long-term effects on central and effector memory T cells (14), and alteration of the establishment of immunological memory response can be detected for long after delivery (15). Indeed, it is not granted that the exposures to an invading pathogen during pregnancy will trigger a correct and long-lived immunological memory and it has been shown that sub-optimal memory responses are created toward pathogens that infect pregnant woman (16). Suppressive regulatory T lymphocytes (T_reg_) induce an immunologically tolerogenic environment to harbor the fetus (17). Physiologically, T_reg_ frequency increases over the course of the trimesters (18), together with the decrease of co-stimulatory molecules (i.e. B7 family) (19–21). In turn, T cells are less likely to be triggered toward a proinflammatory immune response. Thus, an unbalanced immune response, including the memory compartment, was described to characterize influenza A virus infection during pregnancy (22, 23). No results are nevertheless available on the effects of pregnancy on the development of SARS-COV-2-specific memory lymphocytes. How potent is the immune response against SARS-CoV-2 in pregnant women and how long the specific SARS-CoV-2 immunity might last need to be urgently addressed (24). This is particularly compelling as SARS-CoV-2 vaccinal campaigns are taking place. Surveillance analyses had identified pregnant women as a particularly vulnerable group who is more likely to be hospitalized and to develop severe symptoms (25). Pregnant women were and are nonetheless excluded from clinical trials of the current vaccinal campaign and the vaccine efficacy in this cohort still needs to be assessed (4, 7).

Here, we report the long-term immunological response upon SARS-CoV-2 infection in a group of women who was followed from the delivery to a six-months follow-up. In particular, we investigated the immunological response in terms of immunological memory, specific antibody production and T cell memory subsets, and inflammatory profile. Understanding the immunological dynamics of the immune system in response to SARS-CoV-2 is essential for defining proper obstetric management of pregnant women and setting appropriate vaccinal plans that take care of gender-specific differences, including SARS-CoV-2 convalescent subjects.

## Methods

### Study Population

This is a prospective study that includes 47 pregnant women with SARS-CoV-2-positive first diagnosis when admitted at delivery at Unit of Obstetrics and Gynecology, “L. Sacco” (ASST Fatebenefratelli-Sacco) COVID-19 hub hospital, Northern Italy between March and May 2020.

All patients were admitted with a SARS-CoV-2 infection diagnosis defined by a positive result nasopharyngeal swab. The swab samples were processed by using real-time PCR testing SARS-CoV-2 with the automated ELITe InGenius system and the GeneFinderTM COVID-19 Plus RealAmp Kit assay (ELITechGroup, France), according to manufacturer’s instruction. This assay targets two viral genes: Nucleocapsid protein (N) and Envelope membrane protein (E). The human RNA-dependent RNA polymerase (RdRP) was employed as positive control. All women underwent clinical evaluation of vital signs and symptoms, laboratory analysis and radiological chest assessment at admission was discretional. The therapeutic management was consequently tailored according to the clinical findings and guidelines demographic and anthropometric characteristics, lifestyle habits, medical or obstetric comorbidities. All data were recorded at enrollment through a customized data collection form. All women underwent fetal growth and well-being assessment and obstetric management, as required by local standard protocols.

Monitoring of maternal venous blood sample analysis was performed every 48 hours, including hemoglobin, white blood cells count (WBC), hepatic and renal function tests (alanine transaminase (ALT), aspartate transaminase (AST), creatinine) and inflammation markers (C-reactive protein). Data on COVID-19 treatments, clinical evolution during pregnancy, need for oxygen support and ICU admission were collected. Data on mode of delivery or pregnancy termination, neonatal outcomes, and postpartum maternal clinical evolution were subsequently recorded. A severe subgroup was defined based on: 1) urgent delivery due to maternal respiratory function; 2) ICU/sub-intensive care admission during pregnancy or the post-partum period, or both #1 and #2. All collected data were transferred to an electronic database and data accuracy was independently verified by two study investigators (VMS and FP). Any discrepancy or unclear information was verified with the specific participating center.

### Specimen Collection

The protocol was approved by the local Medical Ethical and Institutional Review Board (Milan, area 1, #154082020). We obtained informed written consent from the mothers to perform the procedure and analysis, in compliance with the Declaration of Helsinki principles.

Blood samples were collected in EDTA at time of delivery (T0), after two weeks (T2), four weeks (T4) and six months (T24). Samples were immediately transferred to the laboratory of Immunology, University of Milan, according to the kind of specimen, to be readily processed. Plasma was separated by centrifugation and conveniently stored for future analyses, as well as peripheral blood mononuclear cells (PBMCs), as previously described (26).

### Antibody Detection

The presence of SARS-CoV-2-specific antibodies was investigated using SARS-CoV-2 IgG and IgM chemiluminescence immunoassay kits on fully automated iFlash1800 analyzer (Shenzen YHLO Biotech Co., Ltd., Shenzen, China): the assay uses nucleocapsid (N) and spike (S) viral proteins as magnetic bead-coating antigens. The value of 10.0 AU/ml was used as the positivity cut-off for IgM, while 7.1 was used for IgG. The limit of detection of this kit is not declared, in agreement with the European Ligand Assay Society. The intra-assay percentage of coefficient variation (%CV) spanned from 2.7 to 5.0 for IgM and the inter-assay %CV spanned from 4.1 to 6.1, while the intra-assay %CV spanned from 2.9 to 4.9 and the inter-assay %CV spanned from 4.0 to 4.9 for IgG.

### Neutralization Assay

2×10^4^ Vero-E6 cells (ATCC^®^ CRL 1586™) were seeded in a 96-well plate in DMEM medium (Euroclone, Milan, Italy) supplemented with 2% FBS, 2.5 mM L-glutamine, 100 U/mL penicillin and 100 μg/mL streptomycin. Plasma was previously incubated at 56° for 30’ in order to heat-inactivate the complement system. Plasma obtained at T24 was employed for this assay. 2000 TCID_50_ of the B.1 (EU) SARS-CoV-2 were pre-incubated at 37° for 30’ with serial dilution 1:2 of patient’s plasma, spanning from 1:20 to 1:1280 in 2% FBS DMEM. Upon incubation, the virus-plasma mix was transferred onto the cells, incubated for 60’ at 37°. Cells were then washed, and the media replenished with fresh 2% FBS DMEM. Optical microscope observation (ZOE™ Fluorescent Cell Imager, Bio-Rad, Hercules, CA, USA) was performed daily to investigate the cytopathic effect. At 72 hours post infection, supernatant was removed, cells fixed in 4% PFA for 10’ at room temperature and then stained with crystal violet (Sigma Aldrich, St. Louis, MO, USA). Each culture condition was run in duplicate, together with an internal control of the virus efficiency of infection.

### Cell Culture

PBMCs were resuspended at the concentration of 1×10^6^ cells/mL in 10% FBS RPMI 1640 medium (Euroclone, Milan, Italy) and subsequently stimulated with 500 μg/mL of S (spike) and N (nucleocapsid) recombinant peptides of SARS-CoV-2 (NovateinBio, Woburn, MA, USA). Unstimulated PBMCs were cultured as control as well. Cells were harvested 10- or 24-hours post-infection (hpi) for RNA or flow cytometric analyses, respectively.

### Flow Cytometry

A complete evaluation of immunological memory concerning the different functional subtypes of SARS-CoV-2-specific circulating T cells was performed at T0, T4, T6 and T24. The immunophenotypic lymphocyte subpopulations were identified by flow cytometry on PBMCs upon SARS-CoV-2 antigen stimulation (*n*=15). The following CD4+ and CD8+ T cell subsets were analyzed: naïve (CD4+ CCR7+ CD45RA+; CD8+ CCR7+ CD45RA+); central memory (CD4+ CCR7+ CD45RA-; CD8+ CCR7+ CD45RA-); effector memory (CD4+ CCR7- CD45RA-; CD8+ CCR7- CD45RA-); terminally differentiated effector memory re-expressing CD45RA (CD4+ CCR7- CD45RA+; CD8+ CCR7- CD45RA+). PBMCs were incubated for 30 minutes at room temperature with the following monoclonal antibodies: anti-human CD4-PeCy7 (eBioscience, San Diego, CA, USA), anti-human CD8-PC5 (Beckman Coulter, California, USA), anti-human CD45RA-FITC (Beckman Coulter, California, USA) and antihuman CCR7-PE (R&D Systems, Minneapolis, USA). Then, the cells were washed with PBS (Euroclone, Milan, Italy) and fixed in 1% paraformaldehyde (Sigma-Aldrich, St. Louis, MO, USA). The lymphocyte population was gated based on its forward and side scatter properties, and further gated for CD4 or CD8 expression; 20,000 events were acquired within the CD4 or CD8 gate. Gallios flow cytometer was employed for acquisition, Kaluza 2.0 software for data analyses (Beckman Coulter, California, USA). Results are expressed in terms of percentages of subpopulations relative to the CD4+ or CD8+ population, respectively.

### Expression Analyses of the Inflammatory Response

Gene expression of 8×10^4^ PBMCs was performed by QuantiGene Plex assay (Thermo Scientific, Waltham, MA, USA), which provides a fast and high-throughput solution for multiplexed gene expression quantitation, allowing the simultaneous measurement of 74 custom selected genes of interest in a single well of a 96-well plate. The QuantiGene Plex assay is hybridization-based and incorporates branched DNA (bDNA) technology, which uses signal amplification for direct measurement of RNA transcripts. The assay does not require RNA purification, nor retro-transcription, with a minimal sample handling. Some of the targets resulted below limit of detection and the arbitrary value of 0 was assigned.

The concentration of 27 cytokines/chemokines was assessed on the plasma specimens collected from all the enrolled subjects at each time-point using magnetic bead-based immunoassays (Bio-rad, CA, USA), according to the manufacturer’s protocol *via* Bio-Plex 200 technology (Bio-rad, CA, USA). Some of the targets resulted to be over-range and arbitrary value of 4000 pg/ml was assigned, while 0 pg/ml was assigned to values below limit of detection.

### Statistics

For the study variables, medians and ranges were reported for quantitative variables, and absolute and relative frequencies for categorical variables. No imputation was made for missing data. All ranges are indicated as minimum–maximum values. t-Test and Mixed-effects model (REML) analysis was used with a *p* value threshold of 0.05. The analyses were performed using GraphPad Prism 8 or SPSS Statistics 26.0.

All the procedures were carried out in accordance with the GLP guidelines adopted in our laboratories.

## Results

### Population

Three patients were classified as severe cases (6%). A radiological confirmation of interstitial pneumonia was obtained on admission or antepartum for all the severe cases and overall in 20 women (43%). Pharmacological treatment during the antepartum period of hospitalization is reported in **Table 1**. Briefly, 30% of the enrolled subjects received Lopinavir/Ritonavir and 23% hydroxycholoroquine. At the time, none of them received Remdesivir, nor combination therapies. In the only severe case of preterm labor, corticosteroids for respiratory distress syndrome prophylaxis were administered.

**Table 1:** Baseline characteristics of the study population on admission.

	Total study population (*n* = 47)
Maternal baseline characteristics	
Maternal age, years, median (range)	30 (20–42)
Pregnant women, *n* (%)	29 (62)
Gestational age at admission, median (range)	38 (6 – 41)
Puerperas*, *n* (%)	18 (38)
Nulliparous, *n* (%)	18 (40)
Multiparous, *n* (%)	29 (60)
Spontaneous conception, *n* (%)	47 (100%)
Pre-pregnancy BMI, kg/m^2^, median (range)	23 (17–37)
Known sick contact, *n* (%)	16 (34)
Smoking habit, *n* (%)	2 (4)
Ethnicity, Caucasian, *n* (%)	27 (57)
Chronic comorbidity, *n* (%)	9 (19)
Flu vaccination in pregnancy, *n* (%)	8 (17)
Ante-partum therapy	
Antibiotic, *n* (%)	12 (26)
Lopinavir/Ritonavir, *n* (%)	14 (30)
Hydroxychloroquine, *n* (%)	11 (23)
Oxygen support without ICU admission, *n* (%)	4 (9)
Positive chest X-ray, *n* (%)	20 (43)
Severe case, *n* (%)	3 (6)
Admission to ICU, *n* (%)	1 (2)

*Early Post-Partum admission.

Maternal and pregnancy outcomes in the study population are reported in **Table 2**. Regarding the mode of delivery, 3 patients (6%) underwent emergency delivery for maternal respiratory indication. Among the severe cases, one needed postpartum admission to the ICU and invasive ventilation for 11 days in total.

**Table 2:** Maternal and pregnancy outcomes in the study population.

	Total study population (n = 47)
Total of deliveries, n (%)	41 (87)
Delivery mode	
Vaginal, *n* (%)	27 (55)
Caesarean section, *n* (%)	14 (30)
GA at delivery, weeks median (range)	39 (25 - 41)
Caesarean section for severe maternal illness related to COVID-19, *n* (%)	3 (6)
Preterm delivery*, *n* (%)	2 (4)
Birth weight, g, median (range)	3100 (1800 – 4165)
Umbilical artery pH, median (range)	7.34 (7.14–7.43)
APGAR score 5’ <7, *n* (%)	2 (4)
Infected neonates, positive, *n* (%)	2 (4)
NICU admission, *n* (%)	1 (2)
Breastfeeding, *n* (%)	24 (51)

*One woman delivered at home a stillborn fetus at 25 weeks of gestation.

### Specific Anti-SARS-CoV-2 Antibody and Neutralization Titer

**Figure 1:**
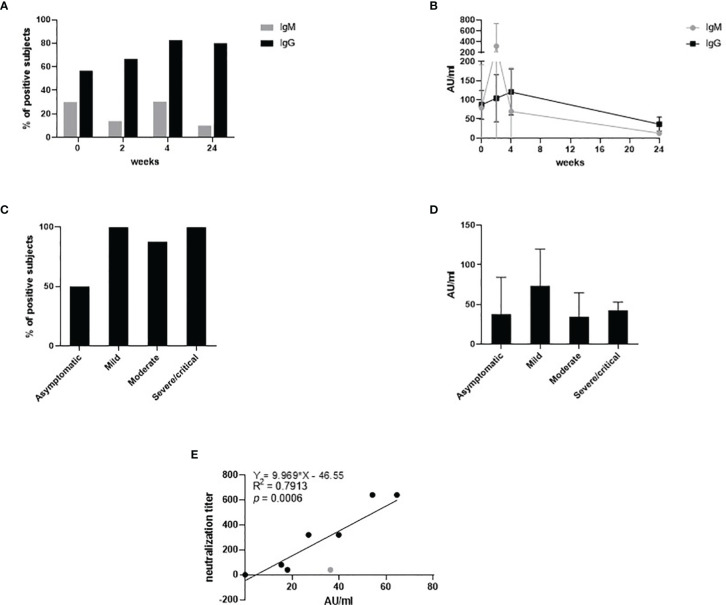
SARS-CoV-2-specific IgM and IgG measured in plasma collected at T0, T2, T4 and T24. The percentage of the positive subjects for IgM (grey) and IgG (black) is depicted throughout the timepoints in **(A).** The antibody titer expressed in AU/ml for IgM (grey) and IgG (black) is depicted throughout the timepoints in **(B)**. The percentage of the positive subjects for IgG at T4 stratified by disease severity is depicted in **(C)**. The antibody titer expressed in AU/ml for IgG at T4 stratified by disease severity is depicted in **(D)**. The neutralization titer related to the IgG titer expressed in AU/ml is expressed in **(E)**.

SARS-CoV-2-specific IgM and IgG were measured in plasma collected throughout the different timepoints as follows: delivery, 2, 4 and 24 weeks post delivery (**Figure 1**).

IgM were detected in 30% of the subject at T0, 14% at T2 and 10% at T24 (**Figure 1A**). IgM peaked sharply at week 2 (T2) with an average value of 320.17 AU/ml (**Figure 1B**). Then, it decreased overtime to 12.8 AU/ml at T24. The percentage of subjects testing positive for IgG increased over-time, to then stabilize at 80% approximately at the 4 and the 24 weeks post-delivery time points (**Figure 1A**). Interestingly, approximately 100% of the mild-to-critical subjects developed IgG during the considered span of time, while IgG became detectable at week 4 only in 50% of the asymptomatic women (**Figure 1C**). IgG peaked at T4 with an average value of 92 AU/ml, and it decreased to 36.48 AU/ml at T4. The titer of the IgG at week 4 showed no significant differences among the subjects stratified by disease severity (**Figure 1D**).

Neutralization capability was titrated in plasma specimens collected at T24 (**Figure 1E**). We chose to evaluate the nAb titer at week 24, as it is more representative of the long-lived immunological memory and it offers an insight on the long-term protection. Results showed that the neutralization titer directly correlated with the IgG titer (R^2 =^ 0.79, *p*=0.0006). Apparently, one subject only did not develop neutralization capability despite the amount of IgG detected was 36.3 AU/ml. By removing this subject (grey dot in **Figure 1E**), the R^2^ would be 0.93 and the statistical significance would drop to <0.0001.

### SARS-CoV-2 Naïve and Memory T-Cell Characterization

**Figure 2:**
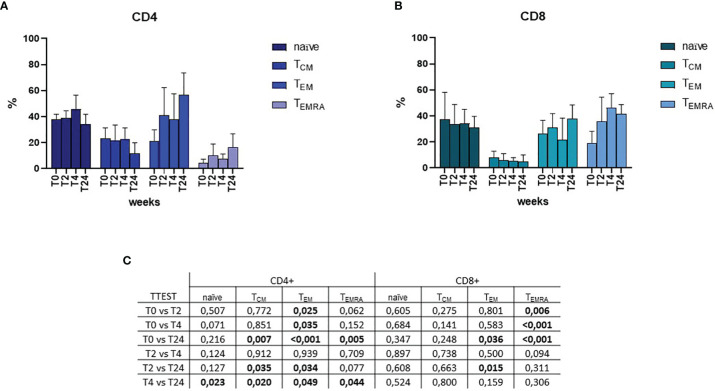
Flow cytometric analyses of memory subsets at T0, T2, T4 and T24 upon SARS-CoV-2 specific *in-vitro* stimulation of PBMCs. Percentages of naïve, central memory (T_CM_), effector memory (T_EM_) and effector memory re-expressing CD45RA (T_EMRA_) T helper CD4+ and T cytotoxic CD8+ are depicted in **(A, B)**, respectively. Mixed-effects model (REML) analysis showed a statistical significance of *p*=0.006 for the time effect on CD4+ cells, and *p*=0.058 for CD8+. Statistical significance obtained by multiple *t* Test analyses is reported in **(C)**. p≤0.05 values are highlighted in bold.

Naïve and memory T cell subsets were assessed by flow cytometry upon SARS-CoV-2 specific *in-vitro* stimulation (**Figure 2**). Throughout the timepoints, naïve cells were stable around a percentage of approximately 40% of the CD4+ T cell and 35% of the CD8+ T cell (**Figures 2A, B**, respectively). The percentage of central memory (CM) T cell significantly decreased over-time from 23% to 11% for CD4+ T cell, while it slightly decreased from 8% at T0 to 6% at T2, and then stabilized over-time for CD8+ T cell. Effector memory T cell (EM) increased over-time in both CD4+ (from 21% at T0 to 56% at T24) and CD8+ (from 26% at T0 to 34% at T24) subsets. Similarly, CD4+ effector memory re-expressing CD45RA (EMRA) T cell increased from 4% (T0) to 13% (T24) for CD4+, whereas CD8+ _EMRA_ increased from 19% (T0) to 41% (T24). Mixed-effects model (REML) analysis showed a statistical significance of *p*=0.006 for the time effect on CD4+ cells, and *p*=0.058 for CD8+. Multiple single *t* Test analyses are shown in **Figure 2C**.

### Inflammatory Response

**Figure 3:**
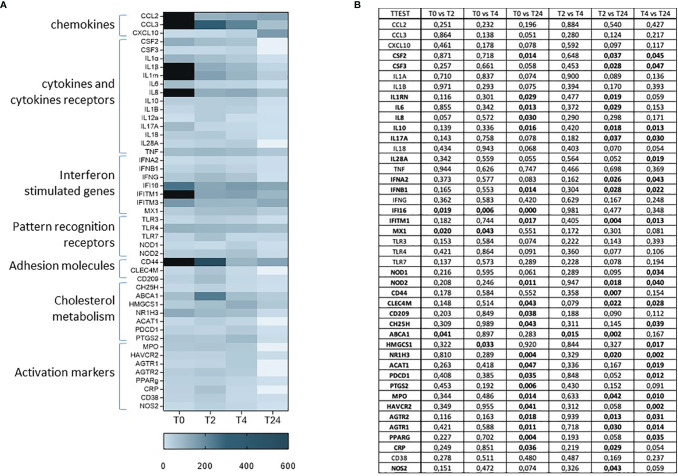
mRNA expression of 74 genes involved in the inflammatory response at T0, T2, T4 and T24 upon SARS-CoV-2 specific *in-vitro* stimulation of PBMCs **(A)**. Gene expression (nfold) is shown as a Colour scale from light to dark blue. Only the most relevant targets are shown in the table. Statistical significance obtained by multiple *t* Test analyses is reported in **(B)**. p≤0.05 values are highlighted in bold.

To determine whether SARS-CoV-2 infection results in a long-term alteration of the immune response in women who became infected during pregnancy, we analyzed the expression of 74 genes involved in the inflammatory response at T0, T4, T6 and T24 upon SARS-CoV-2 specific *in-vitro* stimulation (**Figure 3**). Results showed a generalized immune activation at T0, which quickly decreases over-time (**Figure 3A**), suggesting that the immune activation associated with SARS-CoV-2 infection does not persist over-time. The upregulated genes are involved in different aspects of the inflammatory response and include cytokines and chemokines (CSF2, CSF3, IL1Rn, IL6, IL8, IL17A, IL28), adhesion molecules (CD44, CD209), activation markers (HAVCR2, AGTR1, AGTR2, PPARγ), mediators of the immune response (MPO, CRP and NOS2), downstream signaling molecules of toll-like receptors (NOD1, NOD2), the cholesterol metabolism (CH25H, ABCA1, HMGCS1, NR1H3, PDCD1, PTGS2) and the antiviral interferon stimulated genes (ISG - IFNA2, IFNB, IFI16, IFITM1, MX1). Statistical significances are displayed in **Figure 3B**.

**Figure 4:**
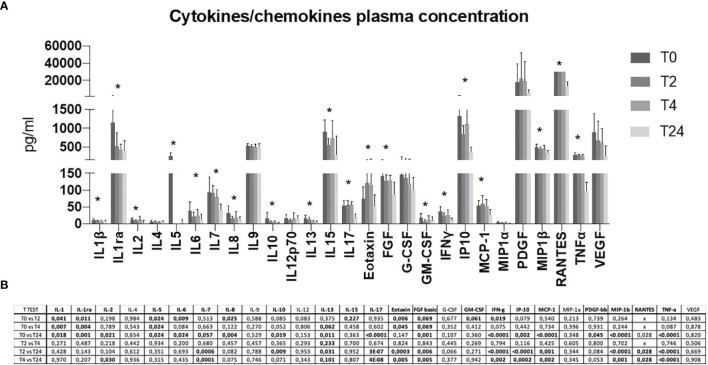
A 27 cytokine/chemokine multiplex array was performed on a maternal plasma at T0, T2, T4 and T24 **(A)**. Protein concentration is shown as pg/ml. Statistical significance obtained by multiple *t* Test analyses is reported in **(B)**. p≤0.05 values are highlighted in bold. * p≤0.05.

A detailed plasmatic cytokine/chemokine profile was assessed. A 27-cytokine multiplex assay was performed on plasma isolated from blood samples (**Figure 4**). Although delivery may modulate the cytokine profile *per* se, overall, the results obtained confirmed what observed at the mRNA expression level. Briefly, proinflammatory antiviral cytokines and chemokines were upregulated at T0 and early time-points, while decreased over-time (**Figure 4A**). This increase was mostly evident for the cytokines IL1β, IL1ra, IL5, IL6, IL7, IL10, IL13, IL15, IL17, Eotaxin, FGF, GM-CSF, IFNγ, IP10, MCP-1, MIP1β, RANTES and TNFα. Statistical significances are displayed in **Figure 4B**.

## Discussion

Herein, we report results obtained by analyzing the potency and the duration of SARS-CoV-2-specific immunological memory in women who became infected during pregnancy; results of a longitudinal assessment of the immunological activation and cytokine profile performed over a 6-month span post-delivery in the same women are presented as well. Our results show that 80% of the women who became infected during pregnancy developed a specific antibody response, consistently with what previously reported for the general population (27, 28). In the analyzed cohort, the antibody titer peaked between week 2 and 4, and it was maintained up to week 24, consistently with how observed in the non-pregnant population (29, 30). Although the lower number of collected samples due to some discontinuity during the follow-up, the neutralization titer significantly correlated with the IgG antibody titer, which is expected but not granted (31). Additional results showed that, while SARS-CoV-2-specifi IgG were present in 50% only of the asymptomatic subjects, strong antibody response was elicited by infection in all mild-to-critical patients. Notably, disease severity did not correlate with the IgG titer and it had been observed in severe COVID-19 patients that the excessive immune response might actually hamper a correct development of a B-cell based memory (32).

Analyses focusing on the development of the anti-SARS-CoV-2-specific immunological memory in this cohort showed that the women enrolled in this study displayed a decreasing percentage of T_CM_, accompanied by an increasing percentage of T_EM_ and T_EMRA_ overtime. T_CM_ cells were predominant in the CD4+ compartment, while T_EM_ in the CD8+ one. Consistent with the literature, this is a sign of a functional and responsive adaptive immune response against SARS-CoV-2 infection (33). As T_CM_ cells express lymph node homing markers, they progressively accumulate in secondary lymphoid organs, where they reside, rather the in peripheral blood (33–35). T_CM_ differentiate into T_EM_, which typically reside in peripheral tissues and patrol the bloodstream, where they display pro-inflammatory effector functions. In this way, T_EM_ cells ensure a strong enhanced recall-response thanks to their increased numbers, higher activation status, reduced stimulatory requirements, more rapid induction of effector functions (35). Further, T_EMRA_ express senescence and exhaustion markers, and display a decreased proliferative capacity, revealing their terminal stage of differentiation from T_EM_ (35–37). Indeed, an increased level of exhaustion markers on T cells is a characteristic trait of SARS-CoV-2 infected patients, even in those displaying mild severity of disease (38–40).

Previously, we assessed the inflammatory response triggered by SARS-CoV-2 infection in pregnant women both at the systemic and placental level, and in their fetuses at the systemic level (41). An intense pro-inflammatory status was detected in maternal and umbilical cord blood in mothers and fetuses. Such inflammatory status was clearly recognizable not only in severe, but even more concerning, also in mild COVID-19 case. A picture of an intense and exaggerated inflammation has been described as being characteristic of severe COVID-19 infection (42) and is clearly detectable in pregnant women during the peri-partum period and soon thereafter (41, 43), fading away after partum and during convalescence. Our results showing that inflammation is greatly diminished within two weeks after delivery confirm previously published observations. Data herein also extend such observations by showing that the inflammatory condition triggered by SARS-CoV-2-specfic stimulation is completely resolved by month six after delivery (44). Although there is no observational study to assess the long-term impact of SARS-CoV-2 infection and the related inflammatory profile during pregnancy on the offspring, strong evidences have been gathered on different viral infections and inflammatory diseases (45–51). Both infections and inflammation during pregnancy may result in direct injury to neurons and neural progenitor cells or indirect injury through activation of microglia and astrocytes, which in turn can trigger cytokine production and oxidative stress. Exposure to infectious or inflammatory agents may perturb neurotransmitter signaling in the developing brain. Detection of such subtle injuries to the fetal brain is difficult, but an increasingly body of evidence links infectious and/or inflammatory events during pregnancy to neuropsychiatric consequences for the child later in life (45–51). SARS-CoV-2-related long-term consequences will need to be scrupulously assessed in the coming years.

Pregnancy represents a peculiar immunological scenario: the mother’s immune system has to balance tolerance towards the fetus with the maintenance of protective responses against invading pathogens (3). The achievement of such balance results in the induction of a ‘window of vulnerability’, that pathogens are more likely to breach (52, 53). This newfound equilibrium has also been shown to hamper the ability to establish an effective long-lived immunological memory towards pathogens encountered in pregnancy (14, 15, 53–57), thus exposing women to an increased risk of reinfection. Such considerations may seriously question the long-term efficacy of the vaccinal campaign for pathogens encountered during pregnancy. Data herein show that pregnancy does not hamper the establishment of an articulate anti-SARS-CoV-2 immunological memory and indicate that virus-specific immune system alterations are quickly reversed upon virus clearance.

Anti-SARS-CoV-2 vaccinal campaigns are being launched all over the world with the shared aim for generating a protective specific immunological memory (58). Although there is not a clear consensus yet about the reciprocal contribution and relative importance of neutralizing antibodies (nAb) *versus* T cell memory, surely both are involved (59). The presence of nAb is enough to completely abrogate viral infectivity *per se* (27). Typically, moderate-to-severe patients develop a high titer of specific antibodies, but it does not correlate with SARS-CoV-2 viral load nor it is predictive of the outcome of the disease (28, 30). Moreover, asymptomatic or pauci-symptomatic patients might not develop specific antibodies not nAb at all, indicating the importance of other mechanisms of immune control, probably T cell-based (59, 60). On the other hand, moderate-to-severe patients consistently develop a robust CD4+ T cell-based response (38, 61). Although too early to assess, some evidences suggest that T cell memory might endure longer than antibody-based immune response in COVID-19 recovered patients (62, 63). Together with the B lymphocytes adaptive immune response (i.e. antibody), the T cell-mediated immunity is proven to be fundamental for an effective resolution of SARS-CoV-2 infection, as often observed in convalescent patients (38, 64–68). Moreover, the mechanisms driving the inauspicious cytokine storm are not fully elucidated so far, especially in the pregnant population. Such syndrome has been described in a number of virus-related severe acute respiratory syndromes, such as MERS, SARS-CoV or seasonal influenza (H5N1 and H1N1) (69). However, in pregnant patients, there are only few data available, and it is absolutely necessary to shed light on a broader picture.

Notably, neither SARS-CoV-2 negative pregnant women nor SARS-CoV-2 positive non-pregnant women were considered appropriate for comparison, and therefore not included in the study design. Our speculations are based on the analyses performed upon SARS-CoV-2 *in vitro* stimulation, and therefore target the antigen-specific immunological response only, and based on the comparison with the state-of-the-art knowledge. These limitations notwithstanding, results herein furnish a novel contribution to a better understanding of SARS-COV-2-specific immunity in women in the particular setting of pregnancy and help in the clarification of SARS-CoV-2 gender issues.

## Data Availability Statement

The raw data supporting the conclusions of this article will be made available by the authors, without undue reservation.

## Ethics Statement

The studies involving human participants were reviewed and approved by local Medical Ethical and Institutional Review Board (Milan, area 1, #154082020). The patients/participants provided their written informed consent to participate in this study.

## Author Contributions

VS and CF conceived the presented idea. VS performed subject enrolment and clinical management, as well as samples collection. CF and MB conceived and planned the experiments. CF, DM, CV, IS, MG, and MS performed and analyzed the experiments. MC, FP, DT, and IC helped with the interpretation of the data. All authors contributed to the article and approved the submitted version.

## Conflict of Interest

The authors declare that the research was conducted in the absence of any commercial or financial relationships that could be construed as a potential conflict of interest.

## Publisher’s Note

All claims expressed in this article are solely those of the authors and do not necessarily represent those of their affiliated organizations, or those of the publisher, the editors and the reviewers. Any product that may be evaluated in this article, or claim that may be made by its manufacturer, is not guaranteed or endorsed by the publisher.
